# Research on Multi-Sensor Data Fusion Based Real-Scene 3D Reconstruction and Digital Twin Visualization Methodology for Coal Mine Tunnels

**DOI:** 10.3390/s25196153

**Published:** 2025-10-04

**Authors:** Hongda Zhu, Jingjing Jin, Sihai Zhao

**Affiliations:** 1 School of Mechanical and Electrical Engineering, China University of Mining & Technology-Beijing, Beijing 100083, China; 2 Huadian Coal Industry Group Digital Intelligence Technology Co., Ltd., Beijing 102488, China; 3 School of Instrumentation Science and Opto-Electronic Engineering, Beijing Information Science and Technology University, Beijing 100192, China

**Keywords:** multi-sensor data fusion, digital twin visualization, real-scene

## Abstract

This paper proposes a multi-sensor data-fusion-based method for real-scene 3D reconstruction and digital twin visualization of coal mine tunnels, aiming to address issues such as low accuracy in non-photorealistic modeling and difficulties in feature object recognition during traditional coal mine digitization processes. The research employs cubemap-based mapping technology to project acquired real-time tunnel images onto six faces of a cube, combined with navigation information, pose data, and synchronously acquired point cloud data to achieve spatial alignment and data fusion. On this basis, inner/outer corner detection algorithms are utilized for precise image segmentation, and a point cloud region growing algorithm integrated with information entropy optimization is proposed to realize complete recognition and segmentation of tunnel planes (e.g., roof, floor, left/right sidewalls) and high-curvature feature objects (e.g., ventilation ducts). Furthermore, geometric dimensions extracted from segmentation results are used to construct 3D models, and real-scene images are mapped onto model surfaces via UV (U and V axes of texture coordinate) texture mapping technology, generating digital twin models with authentic texture details. Experimental validation demonstrates that the method performs excellently in both simulated and real coal mine environments, with models capable of faithfully reproducing tunnel spatial layouts and detailed features while supporting multi-view visualization (e.g., bottom view, left/right rotated views, front view). This approach provides efficient and precise technical support for digital twin construction, fine-grained structural modeling, and safety monitoring of coal mine tunnels, significantly enhancing the accuracy and practicality of photorealistic 3D modeling in intelligent mining applications.

## 1. Introduction

In the field of coal mine safety, real-time monitoring of underground environments has always been a core technical challenge for ensuring workers’ safety and the stable operation of equipment [1]. Traditional visual surveillance systems are often constrained by harsh environmental factors such as poor lighting conditions and dust-filled environments in underground mines, leading to prominent issues like image quality degradation and difficulty in detecting and identifying targets. These limitations make reliable monitoring difficult to achieve. However, underground roadways, as typical confined spaces with relatively fixed structures and distinct geometric features, provide an important breakthrough for innovative surveillance solutions.

Real-scene digital twins are a digital mirroring system of the physical world constructed through high-precision 3D modeling, IoT (Internet of Things) sensing, and real-time data fusion technologies. It dynamically maps personnel, equipment, spatial structures, and operational statuses in real environments, forming a closed-loop system of virtual-real interaction. The core lies in precisely matching and synchronously updating real-world changes in physical space with virtual objects in digital models through multi-source data acquisition methods such as sensor networks, LiDAR (Light Detection and Ranging) scanning, and video surveillance [2,3].

This technology transcends the planar limitations of traditional 2D monitoring systems by presenting underground tunnel structures, equipment layouts, personnel positions, and other elements in a 3D visualization interface. Combined with real-time overlay displays of critical parameters, it provides managers with a “what-you-see-is-what-you-get” holographic perception experience. This reduces the need for personnel to enter hazardous areas, enhances system reliability, and ultimately facilitates the intelligent transformation of mine management from passive response to proactive prevention and control while ensuring safe production.

Although real-scene digital twin technology shows promising application prospects, as previously discussed, underground coal mines face challenges such as insufficient illumination and complex environmental interference. Additionally, effective integration and presentation of multi-sensor information are required. In summary, significant technical challenges remain to be addressed.

Liu Qing et al. proposed a digital twin and collaborative modeling method for the three machines in fully mechanized mining faces, achieving modeling of the three-machine collaborative process [4]. Based on coal mine geological information and intelligent perception data of fully mechanized mining equipment, Fu Dali constructed a remote monitoring system that enables dynamic planning of shearer cutting trajectories [5]. Wang Yan et al. developed a parallel intelligent control method based on the interactive fusion of digital twins and physical systems, realizing virtual tunneling optimization decisions to assist physical tunneling control [6]. Current digital twin research in this field primarily focuses on geometric modeling of virtual entities, with partial studies involving physical, behavioral, and rule-based modeling. However, in practical applications, when real data is fused with virtual entities, the model environment remains essentially a constructed product and cannot reflect authentic roadway environment information, leading to deviations in virtual-real mapping.

In response to the demands and existing challenges of underground monitoring, this paper proposes a real-scene digital twin approach: By introducing navigation and positioning devices to acquire cutting face corner points and planar intersection line coordinates, combined with camera pose relationships to segment real-scene images. LiDAR is integrated to obtain point cloud data, enabling automatic extraction and segmentation of high-threshold targets that reflect three-dimensional object characteristics, followed by projection to achieve 3D scene reconstruction and enhance visualization effects [7,8]. Finally, using homography matrices, 2D images with 3D information are transformed into stereoscopic perspective displays, realizing authentic mapping between virtual digital twins and actual scenes.

## 2. Algorithms and Methodology

### 2.1. Navigation-Based Image Segmentation

In image-based environment modeling approaches, cubic environment mapping is commonly used for overall environment visualization. For example, in the design of reservoir virtual simulation systems, Xue Yutong et al. utilized skybox technology to resample spherical projection panoramas onto the six faces of a cube, achieving reservoir environment visualization [9]. However, this method primarily suits rendering of distant views where image segmentation imposes weak constraints on spatial positioning—segmentation deviations minimally affect overall presentation. In contrast, for near-view environments with complex structures like coal mine roadways, each plane maintains strict spatial correspondence in real space, requiring segmentation to strictly align with actual structures. When cubic environment mapping is applied here, post-mapping images across six faces not only introduce noticeable parallax errors but may also erroneously split a single plane into multiple segments. Consequently, relying solely on cubic environment mapping fails to meet modeling demands for complex near-view environments, necessitating exploration of more rational image segmentation and mapping methodologies [10,11,12].

**Figure 1:**
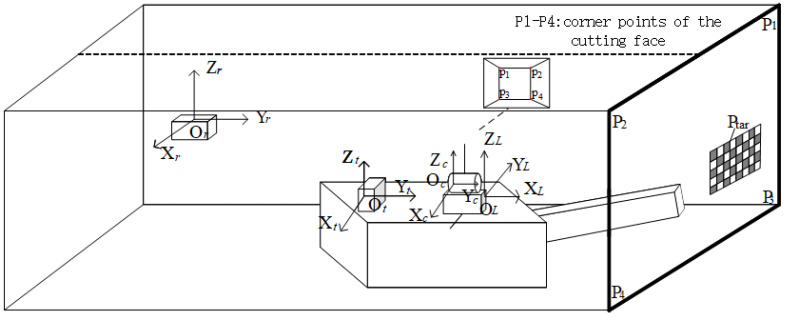
Schematic diagram of the cutting face and multi-sensor on roadheader.

This study incorporates navigation and positioning information from roadheaders to achieve more precise panoramic image segmentation. The positioning system consists of two components: a laser guidance device suspended from the midline of the roadway roof at the rear end, and a pose measurement unit mounted on the roadheader body. The whole navigation and positioning device is manufactured by Beijing BlueVision Technology Co., Ltd., Beijing, China, with attitude accuracy specifications of less than 0.15° in heading, 0.02° in pitch, and 0.02° in roll. As illustrated in Figure 1, the origin of the roadway coordinate system *O_r_* is located on the laser guidance device, with its *X*, *Y*, and *Z*-axes aligned with the lateral direction, heading direction, and vertical direction of the roadway, respectively. This integrated navigation system provides the roadheader’s yaw angle, pitch angle, roll angle, and its coordinates within the roadway coordinate system [13].

To obtain the pixel coordinates of cutting section corner points in the camera coordinate system, pose calibration between the camera and navigation positioning device is first required. The camera is installed such that its field of view fully covers the cutting section, facilitating subsequent image segmentation. A calibration target is mounted at the roadway heading face, with coordinates of each corner point on the target pre-measured in the roadway coordinate system. Let any corner point’s coordinates in the roadway coordinate system be denoted as $P_{tar\_in\_Or}$. By utilizing the camera’s intrinsic parameters, the target’s geometric parameters, and the corresponding pixel coordinates of the corner point’s image formed by the intermediate camera, its coordinates in the camera coordinate system $P_{tar\_in\_Oc}$ can be calculated.

(1) $$\begin{array}{c} P_{tar\_in\_Oc}={\left(R_{O_{c}}^{O_{t}}\right)}^{-1}\cdot \left[{\left(R_{O_{t}}^{O_{r}}\right)}^{-1}\cdot \left(P_{tar\_in\_Or}-T_{O_{t}}^{O_{r}}\right)-T_{O_{c}}^{O_{t}}\right] \end{array}$$

where $R_{O_{t}}^{O_{r}}$ and $T_{O_{t}}^{O_{r}}$ represent the rotation matrix and translation vector from the pose measurement unit coordinate system *O_t_* to the tunnel coordinate system *O_r_*. These can be inversely derived from the heading angle, roll angle, pitch angle, and spatial position coordinates provided by the roadheader navigation and positioning system. $R_{O_{c}}^{O_{t}}$ and $T_{O_{c}}^{O_{t}}$ denote the rotation matrix and translation vector from the intermediate camera coordinate system *O*_c_ to the pose measurement unit coordinate system *O_t_*, which can be calculated by combining multiple corner coordinates and Equation (1).

For the four corners *p_i_* {*i* = 1, 2, 3, 4} in the cutting section, their coordinates in the tunnel coordinate system are denoted as $P_{i\_in\_Or}$, in the pose measurement device coordinate system as $P_{i\_in\_Ot}$, and in the camera coordinate system as $P_{i\_in\_Oc}$. These can be obtained through Equation (2):

(2) $$P_{i\_in\_Oc}=R_{O_{t}}^{O_{c}}\cdot \left(R_{O_{r}}^{O_{t}}\cdot P_{i\_in\_Or}+T_{O_{r}}^{O_{t}}\right)+T_{O_{t}}^{O_{c}}$$

Combined with the camera intrinsic parameters, the corresponding pixel coordinates *p_i_* {*i* = 1, 2, 3, 4} of the corners in the camera image can be further derived, as shown in the following equation:

(3) $$p_{i}=\frac{1}{P_{i\_in\_Oc\_z}}\cdot K\cdot P_{i\_in\_Oc}$$

where *K* is the intrinsic matrix of the camera, and $P_{i\_in\_Oc\_z}$ represents the distance of $P_{i\_in\_Oc}$ along the camera’s *Z*-axis. We can similarly use this approach to verify the correctness of the pose relationship between the camera and the navigation and positioning device. By computing the 3D coordinates of a point on the checkerboard using the above equation, we can project this point onto the camera image and compare its resulting pixel position. Matching points with an error greater than 1 pixel are eliminated. Then the relationship between camera and navigation device is recalibrated.

**Figure 2:**
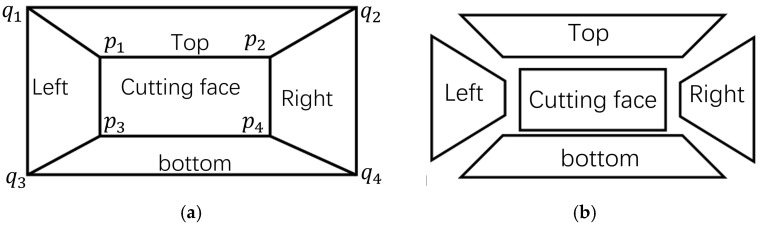
Schematic diagram of roadway image segmentation. (**a**) Cutting face of tunnel, (**b**) segmentation by corner point.

As illustrated in Figure 2, to achieve accurate image segmentation, besides obtaining the pixel coordinates *p_i_* {*i* = 1, 2, 3, 4} of the section corners, it is also necessary to determine their corresponding pixel coordinates *q_i_* {*i* = 1, 2, 3, 4} at the same depth. In this study, *pi* is defined as the “inner corner” for image segmentation, while *q_i_* is defined as the “outer corner”. By connecting the corresponding *p_i_* and *q_i_*, accurate segmentation as shown in Figure 2b can be realized. The tunnel image is segmentate into five regions based on the connecting lines.

**Figure 3:**
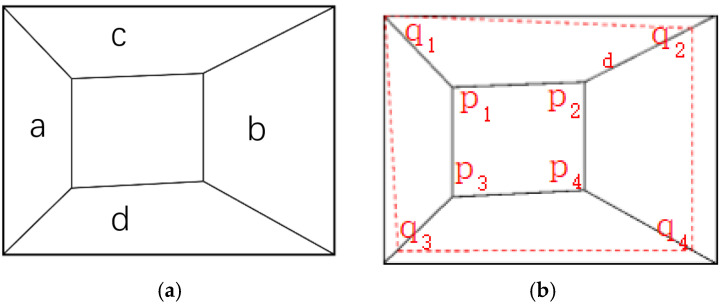
Outer corner point selection. (**a**) Camera view tilts, (**b**) trimming.

Due to the camera’s inability to maintain parallel alignment with the tunnel cutting face during image acquisition, as shown in Figure 3a, when the camera view tilts to the right, region *a* exhibits smaller depth than region *b*, while region *c* shows *a* gradual depth increase from left to right compared to region b. To obtain an approximate cuboid tunnel model, the captured images and point clouds need to be cropped. The known inner corner coordinates in the tunnel coordinate system are $P_{tar\_in\_Or}(x,y,z)$. Since outer and inner corners differ only in the depth direction (*Y*-axis), the outer corner coordinates in the tunnel system can be expressed as $Q_{tar\_in\_Or}(x,y-d,z)$. The corresponding outer corner pixel coordinates *q_i_* in the image are then obtained via coordinate transformation methods. To fully utilize image information, the maximum planar surface *q*_1_*q*_2_*q*_3_*q*_4_ parallel to the cutting face *p*_1_*p*_2_*p*_3_*p*_4_ can be determined using pose data, navigation information, and the pre-calibrated relationship between the camera–LiDAR system and the tunnel coordinate system.

The tunnel image is segmentate into five regions based on the connecting lines between outer corner points and inner corner points.

### 2.2. Point Cloud of Tunnel Plane Segmentation Based on Image Segmentation

**Figure 4:**
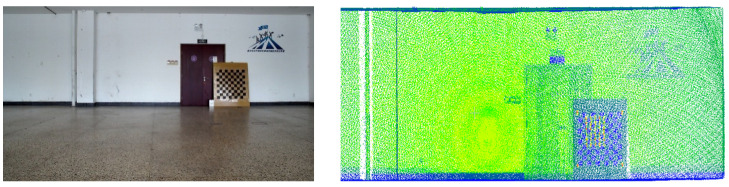
Calibration of camera–LiDAR system.

To visualize three-dimensional information in two-dimensional images, we integrated a LiDAR system with a camera and performed calibration. In the experiments, a Livox-Avia LiDAR (range random error < 2 cm, angular random error < 0.05°, manufactured by Livox Technology Co., Ltd., Shenzhen, China) and a MindVision industrial camera (resolution 1920 × 1080, manufactured by MindVision Technology Co., Ltd., Shenzhen, China) were employed. The Livox-Avia LiDAR operates in a non-repetitive scanning mode, continuously outputting point clouds in dual-echo mode at a rate of 480,000 points per second. Before performing LiDAR-ca-era registration, multiple checkerboard images were captured from different viewpoints using the camera, and the camera intrinsic parameters were estimated with Zhang’s calibration algorithm. Subsequently, multi-view camera images and LiDAR point clouds containing the calibration board were synchronously acquired, from which corner points in the images and echo features in the point clouds were extracted for feature matching. Based on these correspondences, a nonlinear Levenberg-Marquardt optimization algorithm was applied to estimate the six-degree-of-freedom rigid transformation between the two sensors, i.e., the rotation matrix *R* and translation vector *T*. During optimization, the maximum number of iterations was set to 300, and the process was terminated once the residual converged below a threshold of $10^{-6}$. In this way, the extrinsic parameters and relative pose between the LiDAR and the camera were determined [14,15], and the results are shown in Figure 4.

By combining the camera intrinsic matrix *K*, after acquiring tunnel images and point clouds, the tunnel LiDAR point clouds are projected onto the images to establish pixel-wise correspondence between point clouds and their projected positions.

(4) $$\left(\begin{array}{c} X_{L}\\ Y_{L}\\ Z_{L} \end{array}\right)=R\cdot \left(\begin{array}{c} X_{C}\\ Y_{C}\\ Z_{C} \end{array}\right)+T$$

(5) $$\left(\begin{array}{c} u\\ v\\ 1 \end{array}\right)=\frac{1}{z}\cdot K\cdot \left(\begin{array}{c} X_{C}\\ Y_{C}\\ Z_{C} \end{array}\right)$$

where $\left(X_{L},Y_{L},Z_{L}\right)$ represents the 3D coordinates of the tunnel LiDAR point cloud in the LiDAR coordinate system. $\left(X_{C},Y_{C},Z_{C}\right)$ denotes the 3D coordinates of the same point in the camera coordinate system. Equation (4) transforms coordinates from the LiDAR system to the camera system. (*u*, *v*) are the pixel coordinates in the image, calculated via Equation (5) by projecting 3D points from the camera coordinate system to 2D image space.

**Figure 5:**
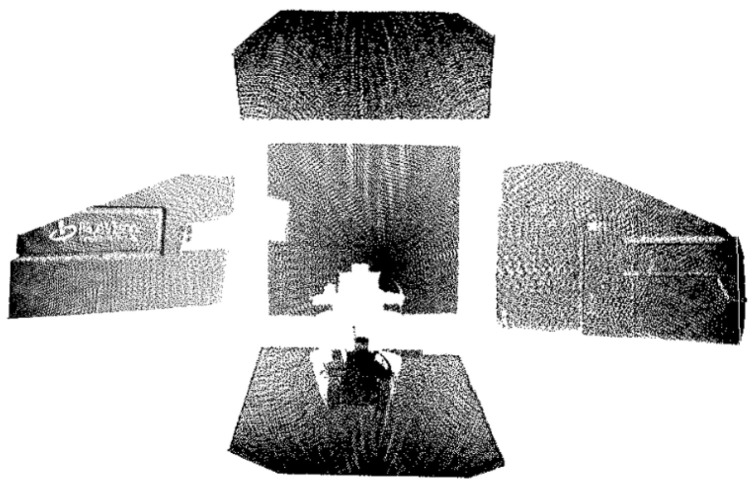
Point cloud segmentation.

Based on this mapping relationship, the segmented images are used to synchronously segment the point cloud data, as shown in Figure 5. Subsequently, the overall geometric dimensions (depth *d*, width *w*, height *h*) of the segmented point cloud are extracted to provide data support for subsequent 3D model reconstruction.

### 2.3. Point Cloud Extraction of Objects in Tunnels Based on Regional Growth

To accurately extract structural features in tunnel environments, this paper employs a region-growing algorithm to perform plane detection on point clouds acquired by LiDAR, using the angle between point cloud normal vectors as the discriminant constraint for region growth. The determination of the key threshold for this constraint relies on the optimization evaluation of point information entropy.

#### 2.3.1. Normal Vector Angle Threshold Calculation and Estimation

In the specific implementation, a local neighborhood is first constructed for each point in the point cloud. Experimental validation in this study shows that using 50 neighboring points effectively balances the preservation of local details with the suppression of noise, ensuring the robustness and accuracy of normal vector and angle calculations. Therefore, this value is selected as the default parameter in the algorithm. Based on the neighborhood point set, a covariance matrix is constructed, and the normal vector direction of the point is obtained through eigenvalue decomposition [16,17,18,19]. The eigenvector corresponding to the smallest eigenvalue is the normal vector. Meanwhile, the curvature estimate of the point is calculated using the eigenvalues, with smaller curvature values indicating flatter regions. Region growth is then performed by sequentially selecting points with the smallest curvature as seed points, using the normal vector angle threshold as a constraint to detect planes in the point cloud.

In tunnel environments, structural features such as ventilation ducts typically exhibit higher curvature than smooth surfaces like walls. During region-growing-based plane detection, the setting of the normal vector angle threshold significantly impacts recognition performance: if the threshold is too large, non-planar regions with high curvature may be misclassified as planes; if it is too small, it may fail to fully capture the entire extent of feature objects. Therefore, selecting an appropriate threshold is crucial for accurately distinguishing smooth areas from complex structural features.

To address this, this paper introduces point information entropy as a criterion for threshold optimization. Information entropy can measure the degree of uncertainty or disorder in a system; in the point cloud context, it reflects the complexity and dispersion of local normal vector distributions. Specifically, for a discrete probability distribution, the entropy is defined as [20]:

(6) $$H(p)=-\sum_{i=1}^{n}p_{i}\log_{2}p_{i}$$

where *pi* denotes the probability of the *i*-th event. In point cloud processing, higher entropy values correspond to regions with relatively uniform normal vector distributions, typically indicative of flat surfaces, whereas lower entropy values often occur in areas with significant geometric variations, such as edges, corners, or other feature-rich regions.

To determine the optimal angle threshold, all normal vector angles are sorted in ascending order, and each angle is sequentially used as a candidate threshold to classify all point pairs into two categories: those less than or equal to the threshold are considered potential “intra-plane” pairs, while those greater than the threshold are regarded as “inter-plane” pairs. The entropy values of these two subsets are then computed separately. If the threshold is too small, the first subset will have high purity and low entropy, but the second subset will contain a mixture of point pairs that should belong to the same plane and those representing true features, resulting in high disorder and elevated entropy. Conversely, if the threshold is too large, the first subset may incorporate noise, increasing its entropy, while the second subset, though pure, remains high in entropy, potentially leading to a suboptimal total entropy.

To balance the segmentation quality between these two types of regions, this paper aims to minimize the weighted total entropy as the objective function to identify the best threshold:

(7) $$H_{total}=w_{1}H(D_{1})+w_{2}H(D_{2})$$

**Figure 6:**
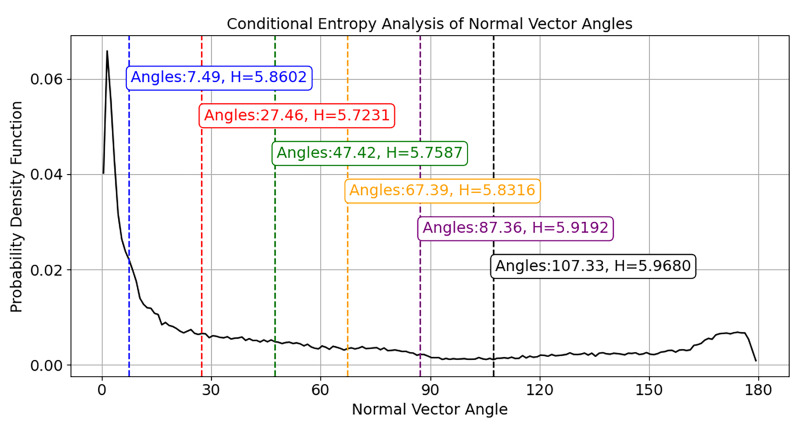
Optimal curvature threshold determination via conditional entropy analysis.

Here, *D*_1_ and *D*_2_ represent the two points sets divided according to the current threshold, and *w*_1_ and *w*_2_ are their corresponding weights, generally taken as the proportion of points in each subset relative to the total. This weighted total entropy reflects the efficacy of the current threshold in distinguishing between intra-plane and inter-plane point pairs. Ultimately, the threshold that minimizes *H_total_* selected as the constraint for the normal vector angle in the region-growing algorithm, enabling more accurate and robust plane extraction. Figure 6 illustrates the optimal curvature threshold determined via conditional entropy analysis of normal vector angles, where the red dashed line corresponds to the threshold yielding minimal entropy *H*. All other thresholds (colored dashed lines) result in higher entropy values. Using this optimal angle threshold as the region growing constraint enhances plane segmentation accuracy and stability.

#### 2.3.2. Point Cloud Segmentation and Extraction Based on Normal Vector Angle Threshold

After obtaining the optimal threshold *ϕ*, points with minimal curvature are selected as seed points for region growing. The angle between the seed point’s normal vector *n_i_* and its neighborhood points’ normal vectors *n_j_* is calculated. If *θ_ij_* ≤ *ϕ*, the neighborhood point is incorporated into the current region and updated as a new seed for further growth. This process repeats until all seeds in the current region complete growth, forming a complete smooth region.

To extract tunnel features, information entropy is employed to evaluate regions by quantifying their internal structural complexity. Based on multiple sets of repeated experiments involving point cloud samples collected from tunnel walls, the calculated entropy values were found to be highly concentrated. Points with information entropy less than 0.3 accounts for 99.7% of the total data set. Accordingly, a statistically significant threshold of *H* < 0.3 was selected to identify planar regions. Areas with low entropy (i.e., *H* < 0.3) exhibit consistent normal vectors, typically corresponding to planes or regular surfaces, whereas regions with high entropy (i.e., *H* ≥ 0.3) show dispersed normal vectors, indicating complex geometries that are classified as feature objects. This statistically validated method enables accurate and stable extraction of objects from the point cloud.

### 2.4. 3D Reconstruction Based on Point Cloud Projection

After identifying high-curvature surfaces, these regions are extracted for further processing. To visualize three-dimensional information on 2D images, the extracted object point clouds are first processed to form continuous depth-reflective surfaces. This study employs a scattered point interpolation method to construct an interpolation function *F* (*x*, *y*). Taking ground objects as an example, the depth coordinate *z* is set as the output of the interpolation function, while 2D planar coordinates (*x*, *y*) serve as inputs. Within a given (*x*, *y*) range, a regular grid point set (*X*, *Y*) is generated, and corresponding *Z* values are calculated using the interpolation function. Adjacent grid points are then connected to form a regularized surface mesh, achieving the conversion from discrete point clouds to continuous surfaces.

Finally, leveraging the pre-calibrated pose relationship between LiDAR and camera, the generated 3D mesh surface is projected onto the segmented 2D image plane. This results in a 2D image with grid lines, effectively fusing 3D surface information with the 2D image.

### 2.5. 3D Viewpoint Conversion

In actual coal mine tunnel images, limited by camera installation positions and shooting angles, the perspectives of different regions vary. However, the model requires orthographic planar images for accurate matching and fusion with the 3D model. To achieve this, inclined side-view images must undergo viewpoint correction and be transformed into stereoscopic orthographic views.

The orthographic view sizes for different regions correspond to the geometric dimensions obtained from point cloud segmentation.

**Figure 7:**
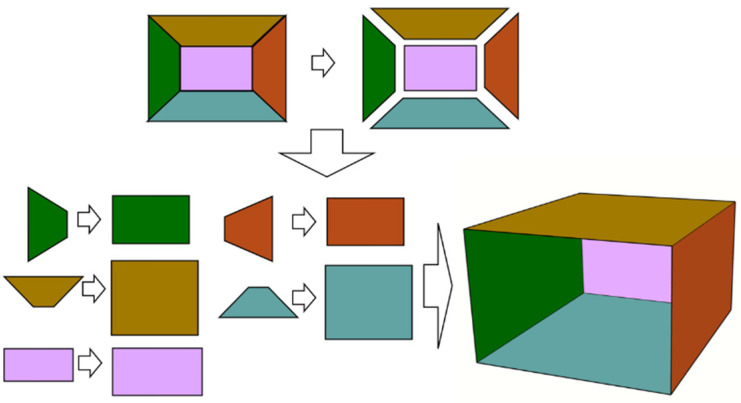
Schematic of tunnel viewpoint transformation.

As shown in Figure 7:

(1) The left and right sidewall orthographic views have dimensions based on the actual tunnel depth *d* and height *h*.
(2) The roof and floor orthographic views use the tunnel width *w* and depth *d*.
(3) The cutting face orthographic view adopts the tunnel width *w* and height *h*.

Using pixel coordinate correspondences between source image corners (*u*, *v*) and target orthographic plane corners (*u*′, *v*′), four pairs of corresponding points are established. The homography matrix *H* is solved via least squares, with the transformation expressed as:

(8) $$s\left[\begin{array}{c} u\prime \\ v\prime \\ 1 \end{array}\right]=H\left[\begin{array}{c} u\\ v\\ 1 \end{array}\right],H=\left[\begin{array}{ccc} h_{11} & h_{12} & h_{13}\\ h_{21} & h_{22} & h_{23}\\ h_{31} & h_{32} & h_{33} \end{array}\right]$$

Here, *s* is the homogeneous coordinate scale factor. To resolve scale ambiguity, we constrain *h*_33_ = 1. Expanding Equation (8) for each correspondence yields two linear equations per point:

(9) $$u_{i}^{\prime }\left(h_{31}u_{i}+h_{32}v_{i}+h_{33}\right)=h_{11}u_{i}+h_{12}v_{i}+h_{13}$$

(10) $$v_{i}^{\prime }\left(h_{31}u_{i}+h_{32}v_{i}+h_{33}\right)=h_{21}u_{i}+h_{22}v_{i}+h_{23}$$

for *i* = 1, 2, 3, 4. These equations are assembled into matrix form:

(11) $$A_{i}=\left[\begin{array}{ccc} \begin{array}{ccc} u_{i} & v_{i} & 1\\ 0 & 0 & 0 \end{array} & \begin{array}{ccc} 0 & 0 & 0\\ u_{i} & v_{i} & 1 \end{array} & \begin{array}{ccc} {-u}_{i}^{\prime }u_{i} & {-u}_{i}^{\prime }v_{i} & {-u}_{i}^{\prime }\\ {-v}_{i}^{\prime }u_{i} & {-v}_{i}^{\prime }v_{i} & {-v}_{i}^{\prime } \end{array} \end{array}\right]$$

Defining the parameter vector:

(12) $$h={\left[\begin{array}{ccc} h_{11} & h_{12} & \begin{array}{ccc} h_{13} & h_{21} & \begin{array}{ccc} h_{22} & h_{23} & \begin{array}{ccc} h_{31} & h_{32} & h_{33} \end{array} \end{array} \end{array} \end{array}\right]}^{T}$$

For all corresponding points, adding *A*_i_ vertically to form matrix *A* yields a homogeneous linear system of equations

(13) Ah=0

Perform singular value decomposition on *A*:

(14) $$A=U\Sigma V^{T}$$

The solution *h* is taken as the right singular vector corresponding to the smallest singular value (last column of *V*). Reshaping *h* into a 3 × 3 matrix yields the homography *H*. This matrix is then applied to perform viewpoint transformation on segmented images.

### 2.6. Tunnel 3D Modeling and Texture Mapping

Based on the segmented point cloud data, the tunnel’s spatial dimensions (length, width, height) can be acquired to constrain the geometric construction of the 3D model. After completing the 3D model reconstruction, real image data must be mapped onto the model surface for visualization. This study employs UV coordinate mapping technology:

(1) UV Coordinate Assignment: each vertex of the 3D model is assigned normalized 2D coordinates (*u*, *v*) within the range [0, 1], corresponding to positions on the texture image.
(2) Texture Projection: the preprocessed, segmented, and viewpoint-transformed images are projected onto the 3D model surface according to the (*u*, *v*) coordinates of the vertices.
(3) Color Interpolation: the color of each triangular patch vertex is interpolated using the pixel values at the corresponding (*u*, *v*) coordinates in the texture image, ensuring texture continuity and seamless adhesion to the model surface.

This process achieves high-fidelity visual reconstruction and multi-view display of the tunnel environment, enabling accurate fusion of 3D geometry and real tunnel environment texture information.

## 3. Experimental Verification and Results

### 3.1. Simulated Scene Experiment

**Figure 8:**
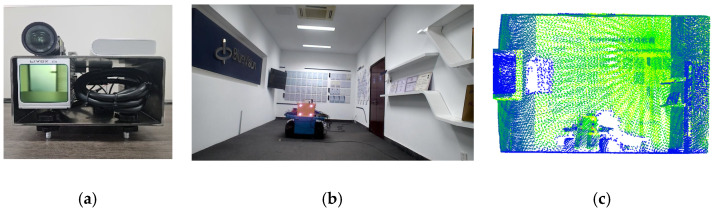
Schematic of the constructed simulation experiment system. (**a**) Camera–LiDAR system, (**b**) simulated tunnel schematic, and (**c**) simulated tunnel point cloud.

To validate the effectiveness of the proposed method, a LiDAR-camera fusion measurement system was designed and constructed. Referencing actual underground excavation face scenarios, an indoor simulated tunnel environment was built to acquire point cloud data from the simulated tunnel, as shown in Figure 8.

Figure 8a presents the camera–LiDAR assembly diagram. In Figure 8b, the corridor represents the simulated excavation tunnel, with the front wall serving as the cutting face. A blue trolley in the tunnel center simulates the roadheader, equipped with a navigation and positioning system that provides real-time position and attitude information of the roadheader within the tunnel. Following real-world conditions, the laser guidance device (origin of the tunnel coordinate system *O_t_*) in the navigation system is located approximately 18 m behind the machine body in the tunnel center. The camera–LiDAR system is positioned 6 m behind the cutting face, with known coordinate values for the simulated tunnel cutting face corner points in *O_r_*.

**Figure 9:**
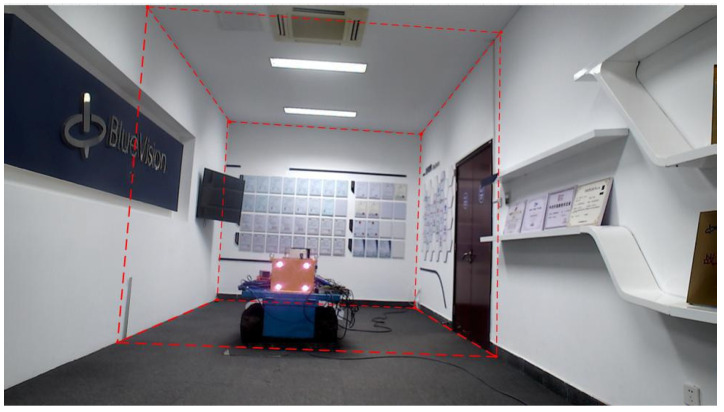
Schematic of inner/outer corner points at optimal distance in virtual tunnel.

Using the method described above, the 3D coordinates of the inner corner points of the cutting face could be obtained via the navigation and positioning device. The outer corner points could be determined by calculating the largest plane in the image that is parallel to the cutting face, and the optimal distance is defined as the depth difference between the inner and outer corner points. The outer corner points computed in the simulated tunnel are highlighted in red in Figure 9, providing an intuitive visualization of their positions.

**Figure 10:**
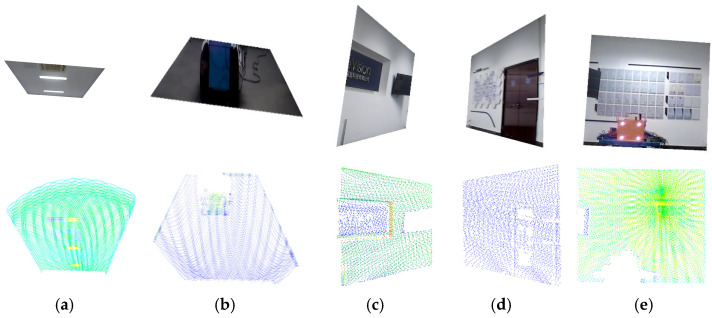
Simulated experiment: image–point cloud plane segmentation results. (**a**) top, (**b**) bottom, (**c**) left, (**d**) right, and (**e**) cutting face.

Using lines connecting the inner and outer corner points from Figure 9, the simulated tunnel image was segmented. Simultaneously, the point cloud was segmented utilizing the pose relationship between the camera and LiDAR, which also allowed the simulated tunnel to be measured with a depth of 4.2 m, a width of 3.5 m, and a height of 3 m. The results are shown in Figure 10.

**Figure 11:**
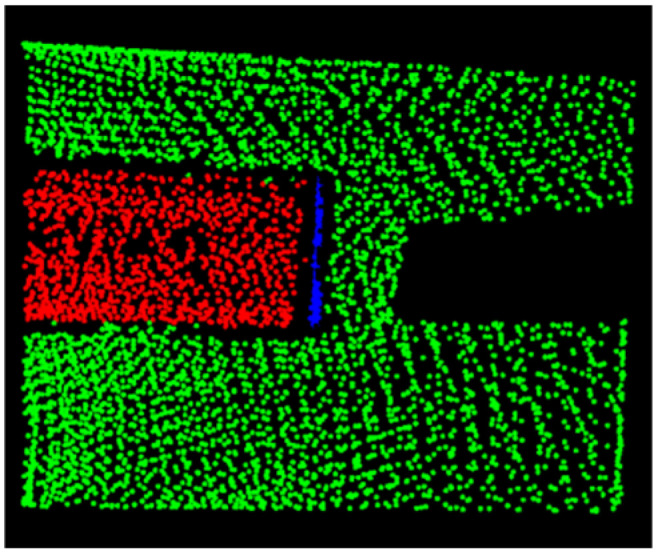
Schematic diagram of simulated tunnel region growth recognition.

In the simulated coal mine tunnel environment, the normal vector angle threshold was determined using the Information Entropy Method for the segmented point cloud planes. Based on this threshold, the Region Growing Algorithm was applied to perform plane segmentation. Subsequently, the total entropy of each segmented plane was recalculated, and regions with total entropy exceeding 0.3 were identified. The final detection results are shown in Figure 11, where the red regions represent the detected feature object planes, while different colors indicate distinct segmented planes. It can be observed that the feature objects on the left side of the simulated tunnel were fully detected, and no segmentation errors or over-segmentation occurred.

**Figure 12:**
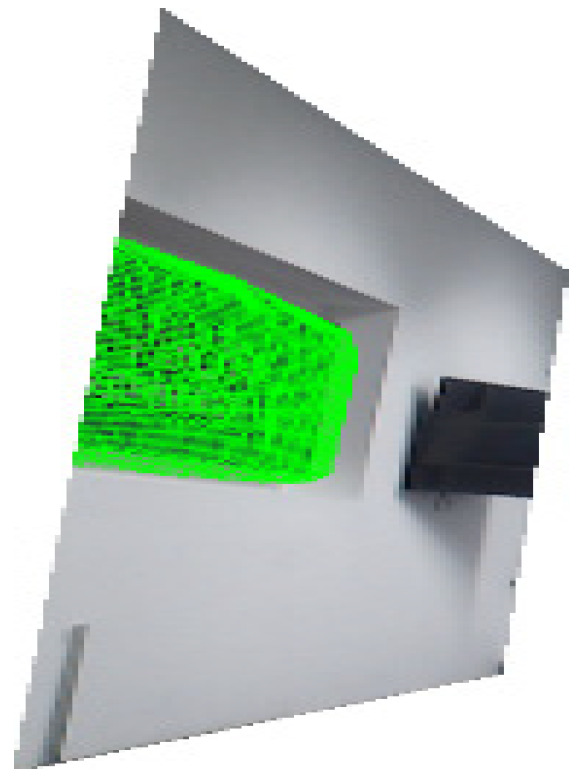
Schematic diagram of feature object grid projection.

Subsequently, mesh fitting was applied to the identified feature objects to generate surface models, which were then projected onto the segmented images. The projection results are shown in Figure 12, where the green meshes represent the 3D projection effects of the point clouds. When overlaid on the images, these meshes clearly demonstrate the three-dimensional structural characteristics of the objects.

**Figure 13:**
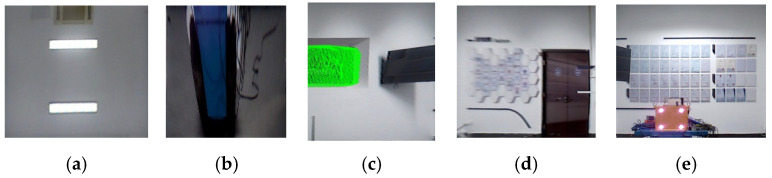
Orthographic view processing results for simulated tunnel planes. (**a**) Top, (**b**) bottom, (**c**) left, (**d**) right, and (**e**) cutting face.

Based on the point cloud data, the geometric dimensions (length, width, height) of the simulated tunnel image were extracted to calculate the homography matrix H required for camera viewpoint conversion. The transformed image results are shown in Figure 13.

**Figure 14:**
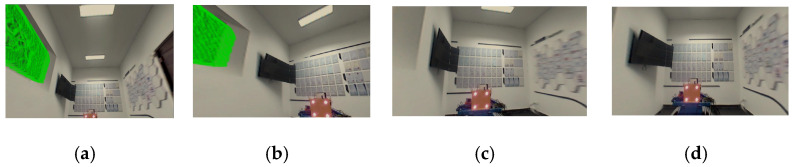
Multi-view visualization of texture mapping results. (**a**) Bottom view, (**b**) left view, (**c**) right view, and (**d**) front view.

Finally, UV texture mapping was performed between the orthographic views and the generated 3D model. After mapping, the model was visualized from multiple perspectives—bottom view, left rotated view, right rotated view, and front view—with the results shown in Figure 14.

### 3.2. Real-Scene Tunnel Experiment

**Figure 15:**
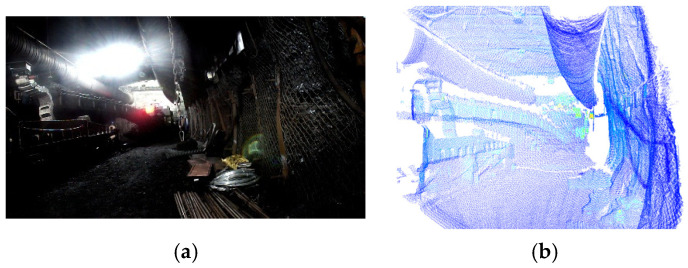
Real-scene tunnel images and point clouds. (**a**) Real-scene tunnel image, (**b**) real-scene tunnel point cloud.

The same methodology was applied to acquire synchronous images and point clouds in a real coal mine tunnel environment. The acquisition results are presented in Figure 15.

**Figure 16:**
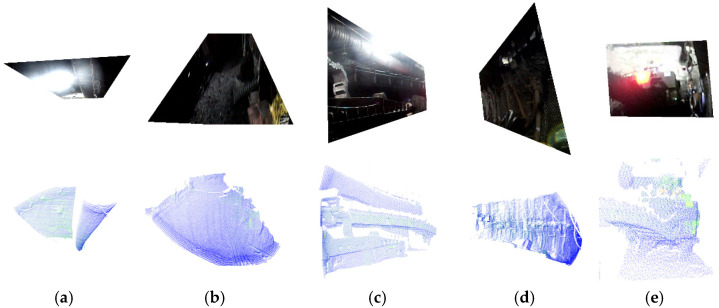
Real-scene tunnel image and point cloud plane segmentation results. (**a**) Top, (**b**) bottom, (**c**) left, (**d**) right and (**e**) cutting face.

Based on the derived inner and outer corner point information, regional partitioning was performed on the actually acquired images and point clouds. The segmentation results are shown in Figure 16, demonstrating that all tunnel planes (roof, floor, side-walls, and cutting face) were completely identified and successfully segmented in both image and point cloud data. In addition, the tunnel model was measured to have a depth of 26.15 m, a width of 6 m, and a height of 3.5 m.

**Figure 17:**
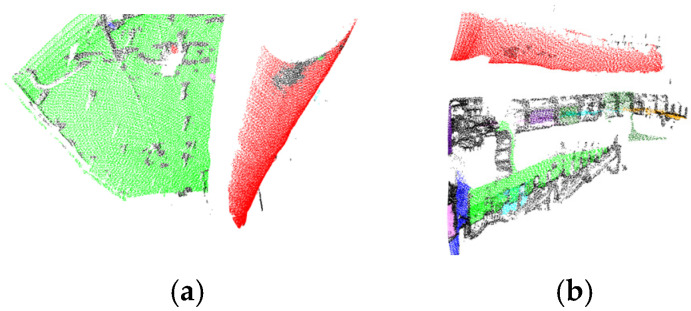
Schematic of region growing plane recognition, (**a**) top, (**b**) left.

In the real-scene tunnel environment, the same Information Entropy Method was applied to determine the Normal Vector Angle Threshold for the segmented point cloud planes. Based on this threshold, the Region Growing Algorithm was implemented. Subsequently, the total entropy of each identified plane was further calculated, and regions with total entropy greater than 0.3 were filtered out. The detection results are shown in Figure 17, where the red regions correspond to the detected feature object planes, while different colors represent the independently segmented planes. It can be observed that the ventilation ducts on the roof and left rib were fully detected, without any mis-segmentation or over-segmentation.

**Figure 18:**
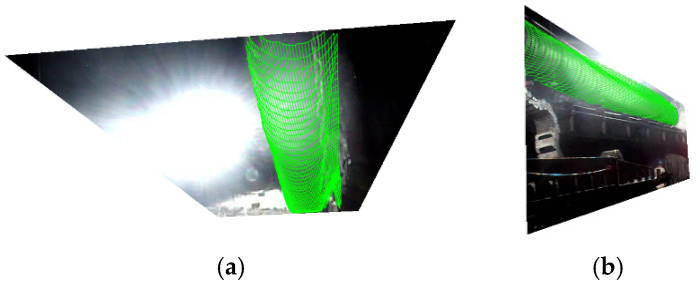
Schematic of ventilation duct mesh projection (**a**) top, (**b**) left.

On this basis, mesh fitting was applied to the segmented ventilation duct point clouds to generate surface models, which were projected onto the segmented images. The projection results are shown in the Figure 18, where green meshes represent 3D projection effects of the point clouds, clearly demonstrating the 3D structural characteristics when overlaid on images.

**Figure 19:**
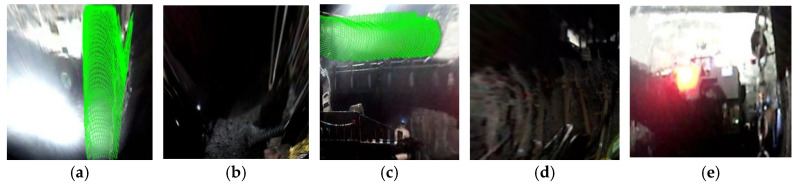
Orthographic view processing results for real-scene tunnel planes. (**a**) Top, (**b**) bottom, (**c**) left, (**d**) right, and (**e**) cutting face.

After perspective conversion processing for all segmented results, the visualized outcomes are shown in the Figure 19.

**Figure 20:**
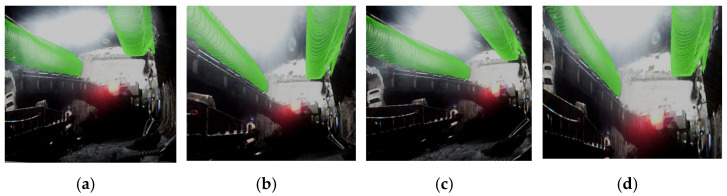
Multi-view points visualization of texture mapping results for actual tunnel. (**a**) Viewpoint 1, (**b**) Viewpoint 2, (**c**) Viewpoint 3, and (**d**) Viewpoint 4.

Based on the actual geometric dimensions obtained from point cloud segmentation, a 3D model was constructed. UV texture mapping was performed between the orthographic view images (after perspective conversion) and the model. Post-mapping visualization from multiple perspectives is shown in Figure 20. The model accurately presents the spatial stereoscopy of ventilation ducts, with clear planar structures (roof, left/right sidewalls) and preserved details. The overall tunnel morphology and spatial layout are faithfully reproduced, achieving a digital twin visualization for subsequent analysis and simulation.

During multi-view visualization performance testing, the system demonstrated excellent real-time performance and stability. Specifically, the average frame rate remained stable at approximately 60 fps during dynamic viewpoint switching, and the average response latency was less than 10 ms. These performance metrics fully meet the general standards for real-time interactive visualization systems, delivering a smooth and stutter-free operational experience for users.

Compared to existing and newly emerging digital twin display solutions, this system innovatively integrates a high-precision navigation and positioning system, enabling intelligent segmentation of monitoring images and enhanced target display, which significantly improves image recognition and positioning accuracy in complex scenarios. To address challenges commonly encountered in coal mine tunnel environments—such as low light, high noise, and insufficient visual information—the system incorporates LiDAR-based multimodal sensing technology. Through deep fusion of camera, LiDAR, and navigation positioning units, it effectively captures and supplements high-resolution 3D point cloud information that images alone cannot adequately represent. Building on this, the system achieves multi-source data-coordinated 3D reconstruction and real-time rendering, maintaining stable and precise scene perception and visualization performance even under extreme optical conditions, demonstrating notable technical advancement and environmental adaptability.

The method proposed in this study not only meets industrial application standards in terms of performance metrics but also represents an innovative approach in system integration, providing a reliable solution for the practical application of visualization and digital twin systems in underground spaces.

## 4. Development of Real-Scene Digital Twin Software for Coal Mine Tunnels

**Figure 21:**
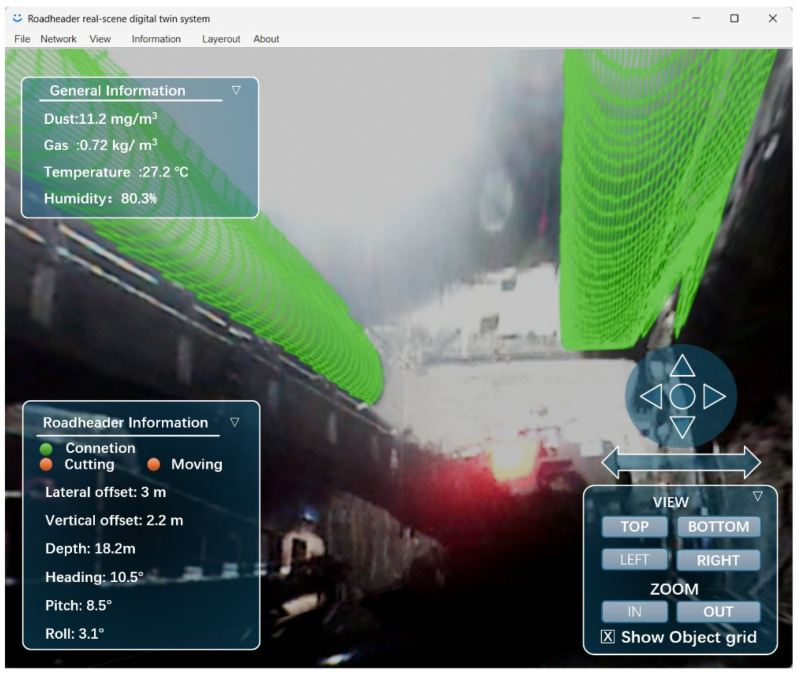
User interface of real-scene digital twin system.

To enhance the practicality and usability of the proposed algorithm, this study developed a dedicated Windows-based software system integrating the core methods, as shown in Figure 21. The software leverages a multi-source data fusion framework to achieve real-time monitoring of tunnel environments, allowing users to interactively visualize and analyze underground conditions through a graphical user interface (GUI). The main functionalities include: (1) dynamic view adjustment through click-and-drag operations, supporting seamless switching between multiple perspectives; (2) real-time display of environmental parameters (temperature, humidity, gas concentrations) synchronized with spatial positioning data; and (3) six-degree-of-freedom pose tracking and visualization of the tunneling machine within the digital twin model. By integrating algorithm processing, data visualization, and human–computer interaction into a unified platform, the software maintains high-precision monitoring capabilities while significantly reducing operational complexity. The system is recommended to be installed locally on a PC with Windows OS above 10, CPU above 12th Intel Core i5 processor, RAM more than 16 GB and a dedicated graphics card to ensure smooth program execution and high-performance graphics processing. This configuration also supports real-time processing of video streams for monitoring the tunneling machine’s main line, as well as handling point cloud rendering and multi-source data fusion tasks. The integrated system has been successfully tested in a pilot coal mine, demonstrating its effectiveness in improving situational awareness and decision-making efficiency for underground operations.

## 5. Conclusions

This paper proposes a cubemap-based 3D reconstruction method for real-scene coal mine tunnels. By mapping acquired images onto the six faces of a cube and performing precise image segmentation using inner/outer corner points, the approach further leverages point cloud region growing and information entropy optimization to identify planar surfaces and high-curvature feature objects. Finally, 3D model construction and UV texture mapping are completed to enable multi-view visualization. Compared to conventional digital twin display solutions, this system innovatively integrates a high-precision navigation and positioning system, enabling intelligent segmentation of monitoring images and enhanced target display, which significantly improves image recognition and positioning accuracy in complex scenarios. Through deep fusion of camera, LiDAR, and navigation positioning units, it effectively captures and supplements high-resolution 3D point cloud information that images alone cannot adequately represent. This method effectively overcomes the limitations of non-photorealistic digital twin representations in coal mine intelligent construction, achieving accurate image-point cloud segmentation and complete feature object recognition in close-range tunnel environments. It provides technical support for tunnel digital twins, fine-grained structural modeling, and safety monitoring applications.

## Data Availability

The raw data supporting the conclusions of this article will be made available by the authors on request.
